# 150 years ago: Schwartze’s 1873 mastoidectomy and its implementation over the following 2 years

**DOI:** 10.1007/s00106-023-01418-3

**Published:** 2024-01-30

**Authors:** Albert Mudry, Stefan K. Plontke

**Affiliations:** 1 School of Medicine, Department of Otolaryngology, Head & Neck Surgery, Stanford University, Stanford, USA; 2 Department of Otorhinolaryngology, Head & Neck Surgery, Martin Luther University Halle-Wittenberg, Ernst-Grube-Str. 40, 06120 Halle (Saale), Germany

**Keywords:** History of medicine, Otologic surgery, Eysell, Mastoid surgery, Surgical technique, Geschichte der Medizin, Ohrchirurgie, Eysell, Felsenbein, Chirurgische Technik

## Abstract

**Background:**

In 1873, Hermann Schwartze and Adolf Eysell described a new surgical technique for treating mastoid disease using a mallet, chisels, and gouges of various sizes instead of trephines or drill instruments also called “modern mastoidectomy.” On the 150th jubilee of this landmark article, we pay tribute by studying the reception and implementation of mastoidectomy in the 2 years following its publication.

**Methods:**

The commentaries published in the otological and medical literature between the second part of 1873 to the end of 1875 were studied with an emphasis on the three specialized otological journals and the otological textbooks that existed during this period.

**Results and conclusion:**

The princeps paper *Ueber die künstliche Eröffnung des Warzenfortsatzes* (“*On the artificial opening of the mastoid process*”) by Hermann Schwartze and Adolf Eysell published in 1873 was rapidly disseminated in the medical literature for nearly 1 year, and then entered a phase of evaluation followed by a phase of extension and implementation, before finding its definitive place in the history of mastoid process surgery.

## Abstract (de)

**Hintergrund:**

Im Jahr 1873 beschrieben Hermann Schwartze und Adolf Eysell aus Halle (Saale) eine neue chirurgische Technik zur Behandlung von Erkrankungen des Warzenfortsatzes unter Verwendung von Hammer, Meißeln und Hohlmeißeln verschiedener Größen anstelle von Trepan- oder Bohrinstrumenten, die auch als „moderne Mastoidektomie“ bezeichnet wird. Anlässlich des 150-jährigen Jubiläums dieses bahnbrechenden Artikels werden im vorliegenden Beitrag die Rezeption und Umsetzung der Mastoidektomie in den 2 Jahren nach seiner Veröffentlichung betrachtet.

**Methoden:**

Dazu wurden die in der otologischen und medizinischen Literatur zwischen der zweiten Hälfte des Jahres 1873 und dem Ende des Jahres 1875 veröffentlichten Kommentare untersucht, wobei der Schwerpunkt auf den 3 otologischen Fachzeitschriften und den otologischen Lehrbüchern lag, die in dieser Zeit existierten.

**Ergebnisse und Schlussfolgerung:**

Das „princeps paper“ mit dem Titel *Ueber die künstliche Eröffnung des Warzenfortsatzes* von Hermann Schwartze und Adolf Eysell aus dem Jahr 1873 fand innerhalb fast eines Jahres eine rasche Verbreitung in der medizinischen Literatur und trat dann in eine Phase der Evaluation ein, gefolgt von einer Phase der Erweiterung und Implementierung, bevor es seinen endgültigen Platz in der Geschichte der Chirurgie des Warzenfortsatzes fand.

## Introduction

**Fig. 1:**
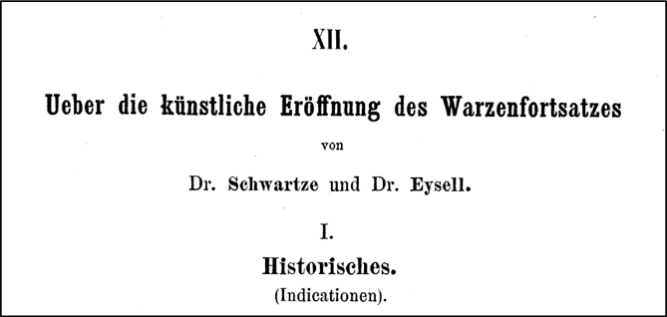
Title page of part I (p. 157) of the seminal work by Schwartze and Eysell [10]

**Fig. 2:**
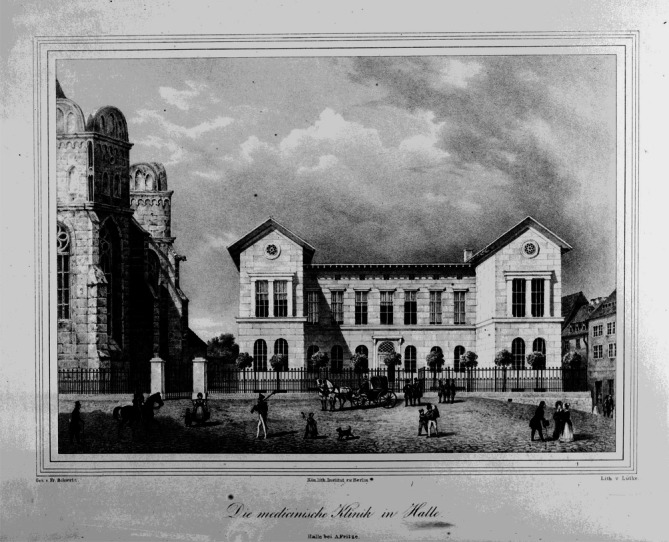
The first hospital at the “Domplatz” (“*Die Medizinische Klinik Halle*” [Department of Medicine Halle]) in the second half of the 19th century. (Reprint with permission by the archive of the Martin Luther University Halle-Wittenberg, UAHW, Rep. 40 VII‑A, Nr. 283. All rights reserved)

Mastoidectomy, in other words, the artificial opening of the mastoid process, is definitively implemented in the field of otology and its technique is described in all books on ear surgery. Unfortunately, the history of mastoidectomy is often not well known. Although the literature on the topic is extensive, it can sometimes be more problematic and obviously inaccurate [1]. After the accidental death of Justus von Berger, physician in the court of Denmark in 1791 [2], the surgical opening of the mastoid, developed in the middle of the 18th century, was largely discredited. In the 1850s new attempts to open the mastoid process were made without much success, and in 1861, Anton von Tröltsch explained that it is necessary to draw again “attention to a discredited and forgotten operation, the opening of the mastoid, and help to give it the place it deserves in surgery” [3]. This paved the way for the two Germans, Hermann Schwartze and his assistant Adolf Eysell in Halle (Saale), to definitively describe and codify this operation in a three-part article titled *Ueber die künstliche Eröffnung des Warzenfortsatzes* (“*On the artificial opening of the mastoid process*”), in the following referred to as the “Schwartze–Eysell publication” (SEP; Fig. 1). The article was published in the otological journal *Archiv für Ohrenheilkunde* (*Archive of Otology*) on June 6, 1873. At the time of the work leading to the publication of the article, the authors were working at the *Medizinisches Krankenhaus am Domplatz* (*Medical Clinic at the Cathedral Square*) in Halle (Saale; Fig. 2). In part three of this article, they described a new surgical technique using a mallet, chisels, and gouges of various sizes [4], and proposed to replace the classic trephine or drill instruments, notably supported by the American Albert H. Buck [5, 6] in an article published 2 months before SEP. Later it was often called “modern mastoid operation” or simply “Schwartze operation” [7]. This new instrumentation enabled the creation of a larger, safer, funnel-shaped opening of the mastoid region down to the antrum, allowing for an expansion of the indication, from mastoid abscess to chronic otitis media. Less than 10 years later, the Austrian Adam Politzer wrote: “It is only within the last twenty years that the real indications have been laid down and the operation perfected from pathological investigations and clinical observations made by von Tröltsch, Forget, Follin, Mayer, Moos, Jacobi, Hartmann, Bezold, and others, but principally by the abundant clinical observations of Schwartze” [8].

The aim of this historical vignette is to celebrate the 150th anniversary of this fundamental article and its quick dissemination in the medical world during the following 2 years after the publication of the princeps paper. The main limitations are the definitive access to all of the related published literature, the voluntary short period of study, and the absence of a comparison with the dissemination of other key publications written at the same time.

## Methodology

Based only on primary sources, we here review the princeps paper and the ensuing commentaries published in the otological and medical literature and in the otological textbooks from the second part of 1873 to the end of 1875. The three specialized otolaryngological journals that existed during this period [9] were collected: the Austro-German *Archiv für Ohrenheilkunde *(*Archive of Otology*, founded in 1864), the Austro-German *Monatsschrift für Ohrenheilkunde *(*Monthly Journal for Otology*, founded in 1867), and the German–American *Archiv für Augen- und Ohrenheilkunde *(founded in 1869) and its English edition *Archive of Ophthalmology and Otology* (the latter do not always contain the same articles!) complemented by the French *Annales des maladies de l’oreille et du larynx* (*Annals of Diseases of the Ear and the Larynx*, founded in 1875). The review was completed by an analysis using the keywords “Schwartze,” “Eysell,” “*künstliche Eröffnung *and *Warzenfortsatz,*” “perforation and mastoid process,” and “*trépanation* and *mastoïde*” of general medical journals publishing otological reports, and otological books, listed and referenced in Google books, and limited to the corresponding time. The resulting references where then submitted to a backward citation search. The analysis of the books was completed by an evaluation of the authors’ personal otological book collection.

## Results

### Schwartze and Eysell’s original publication

The article *Ueber die künstliche Eröffnung des Warzenfortsatzes* (“*On the artificial opening of the mastoid process*”) consists of three parts:

I. History (indications; [10])
II. Anatomy, physiology, pathological anatomy [11]
III. Surgical procedure [12].

#### Part I. History (indications)

Here Schwartze and Eysell give a historical review of isolated case reports of surgery on the mastoid process including the first documented operation by Jean-Louis Petit with a perforator, performed before 1750 but not published until 1774 (after his death) in the *Traité de maladies chirurgicales et des operations*, a rather euphoric case report by Jasser (1776) that probably encouraged the uncritical use of the procedure in the following years, and the first review paper with a collection and evaluation of case reports by Deceimeris (1832), who, however, “with his statistics could not save the operation that had been thrown overboard.” Schwartze and Eysell note that on the basis of the case reports available at that time, the surgical procedure on the mastoid was seen only as an “*indicatio vitalis* … as soon as serious and life-threatening symptoms appear with accumulations of pus in the bony cells of the mastoid,” which they, however, classify as being already too late in terms of preventing a fatal outcome of the disease. Schwartze and Eysell argue—also based on the convictions of Anton von Tröltsch and a large case series of their own observations—for the extension of the indication to cases that do not yet show cerebral symptoms.

#### Part II. Anatomy, physiology, pathological anatomy

In the most extensive second part of the article, Schwartze and Eysell provide an overview of the anatomy of the mastoid and its importance for surgery, its physiological significance, and its pathological anatomy. This is based partly on the available literature and partly on their own observations, physical consideration (or a [thought]experiment), hypotheses, and conclusions. Based on systematic investigations, they derive important conclusions for the surgical procedure, for example, that during an artificial opening of the *processus mastoideus*, the *linea temporalis* “must always lie above the downward and inward guided gouge.” The various complications of inflammatory middle ear and mastoid disease are described in detail, and the authors conclude with the realization that “in many cases nature alone never succeeds in bringing about a cure” and therefore surgical intervention is necessary.

#### Part III. About the surgical procedure

**Fig. 3:**
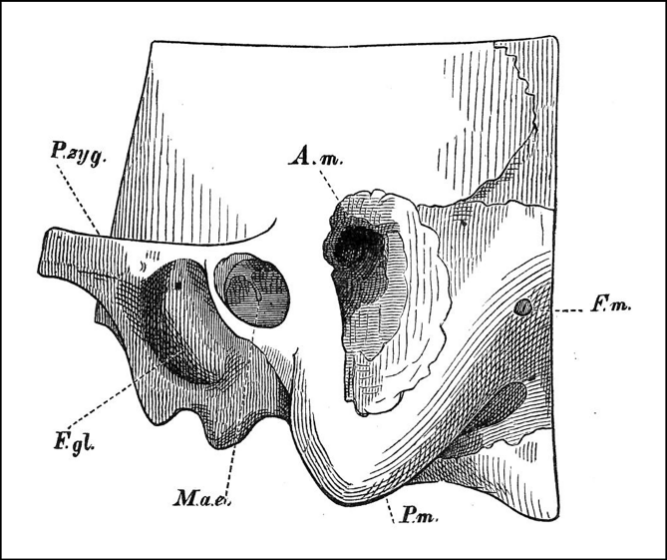
Reprint of Fig. 5, p. 178, from part III of the seminal work of Schwartze and Eysell [12] of a mastoid process completely opened with a chisel. The authors state: “The antrum [lies] somewhat higher than the external auditory canal and therefore, in order to open it, one will have to place the chisel under the *linea temporalis*, which can almost always easily be felt through the uninjured skin, inclined about 45º toward the horizon, and let it act inward, downward and forward. An injury of the dura mater and the transverse sinus is hardly possible, even if one would be forced to penetrate 2 centimeters and deeper. The chisel must never act in the posterior direction …” *A.m.* mastoid antrum, *F.g.l.* glenoid (mandibular) fossa, *F.m.* Foramen of the mastoid vein, *M.a.e.* external auditory meatus, *P. zyg.* zygomatic process, *P.* *m.* mastoid process

Here, Schwartze and Eysell describe the disadvantages of drill-like instruments and advise against the use of perforators and trephines. They recommend the use of gouges and give clear surgical instructions on how to avoid injury to the dura mater and the sinus (Fig. 3). Schwartze and Eysell conclude with a systematic review of all cases published up to 1873, including 17 of their own cases, which corresponds to the largest case series—basically an observational scientific study—published at that time.

### Reception in the medical and otological literature in the second part of 1873

Nearly 6 weeks after the publication of SEP, on July 16, 1873, the American Charles Burnett presented a report on the progress of otology during the 6th Annual Meeting of the American Otological Society [13]. He writes:

“In the interesting paper upon the artificial opening of the mastoid process, by Schwartze and Eysell [sic] of Halle, we find the history of the operation carefully treated […] The historical part of this paper is full of interest […] In the second portion of this valuable paper, the anatomy, physiology, and pathological anatomy are carefully treated […] In alluding to the means for performing the operation, the authors say, that in most cases the outer shell of the skull offers no great resistance to perforation. All kind of instruments may be and have been used with success, but the authors of the paper under consideration give the preference to the gouge. The gouge is preferred because it is applicable on most surfaces, makes less injuries to the adjacent parts, and even accomplishes all that is desired, even in those cases where, in consequence of sclerosis or hypertrophy of the bone, extraordinary difficulties are presented to the operation. Using the gouge, the mastoid antrum can always be found, even where the usual air cells of the process have disappeared.”

On July 30, 1873, the German August Lucae, in discussing “pearly tumor of the petrous bone” in the *Archiv für Ohrenheilkunde*, writes: “I consider the opening of the mastoid process to be a future main indication […] According to Schwartze and Eysell, the safest and most gentle way to do this is to use a gouge” [14].

On October 9, 1873, the American John Orne Green again reported on otology in the *Boston Medical Surgical Journal* (the ancestor of the *New England Journal of Medicine*). He explains that:

“The artificial perforation of the mastoid process is discussed, historically and clinically, by Schwartze and Eysell, and their investigations of the anatomy of the mastoid are of interest, as they have succeeded in describing the relations of that very irregular cavity […] The second part of their paper is occupied with a discussion of the different modes of operation, and the authors prefer to open the bone with the chisel rather than with a borer trephine; no other respects, this section offers nothing new” [15].

In November 1873, the German Friedrich Bezold began a series of three articles in the *Monatsschrift für Ohrenheilkunde* about the perforation of the mastoid process from an anatomical point of view. He explains why SEP is of interest: “The next reason for me to re-examine the anatomical conditions involved in the perforation of the mastoid process was the above-mentioned treatise by Schwartze and Eysell on artificial opening of the mastoid process. This work provides a detailed anatomical description of the development, shape and position of the mastoid cells, which is very grateful, but does not take into account anatomical conditions that are of great importance for perforation” [16]. The second part was published in January 1874 [17], and the third and last part in February 1874 [18]. In this last article, Bezold extensively refers to SEP and explained: “In the work by Schwartze and Eysell listed above, the following rules for perforation are established based on 13 operations carried out by Schwartze himself and taking into account the remaining literature […] This surgical method, which aims to create the perforation canal in such a way that it can always be continued into the mastoid antrum if necessary, has been further developed in particular by Schwartze and Eysell in the manner mentioned above.” Bezold also used the term “Schwartze’s operation.” Eysell commented on these articles in the *Archiv für Ohrenheilkunde* in 1875 [19].

During the fourth quarter of 1873, the French Klein comments, in the *Revue des Sciences Médicales*, on the perforation of the mastoid process: “As perforation as it is practiced is not always without danger, it is useful to study the anatomy of the mastoid process, its physiology, the lesions it may present and finally the mode for the most suitable operation. This is the goal that was proposed by gentlemen Schwartze and Eysell […] The authors give preference to the opening by the gouge and the mallet” [20].

**Fig. 4:**
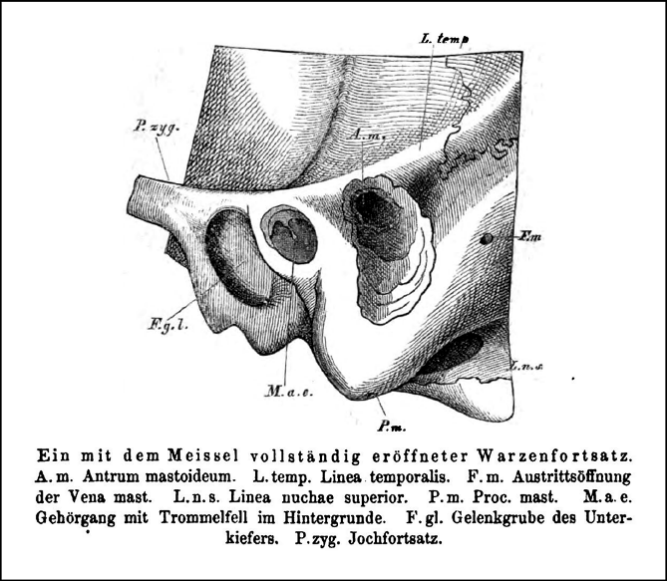
Reprint from an 1873 textbook of otology from Anton von Tröltsch (*Lehrbuch der Ohrenheilkunde*, 5th ed., Leipzig: Vogel, 1873, p. 451) showing a drawing of a completely opened mastoid process with the chisel, very similar to that in the original work by Schwartze and Eysell. *A.m.* mastoid antrum, *F.g.l.* glenoid (mandibular) fossa, *F.m.* Foramen of the mastoid vein, *L.n.* *s.* Linea nuchae superior; *L. temp.* Linnea temporalis, *M.a.e.* external auditory meatus with eardrum in the background, *P.* *m.* mastoid process, *P.zyg.* zygomatic process

The German Anton von Tröltsch, in the 5th edition of his textbook *Lehrbuch der Ohrenheilkunde*, largely discusses SEP. Even more, he nearly uses the same drawing (Fig. 4) of “a complete opened mastoid process with the chisel” and wrote in a note: “I owe this drawing to the kindness of Dr. Eysell” [21]. Interestingly, Tröltsch does not mention the publication in his 1874 book, *The surgical diseases of the ear *[22]. The newest mentioned reference used in this book dated back to 1869, suggesting that the book was written well before SEP, notably because Tröltsch is an editor of the *Archiv für Ohrenheilkunde*, thus certainly aware of SEP at its publication and even before its definitive printing.

The American Daniel Bennett St. John Roosa did not specifically mention SEP in the first three editions of his *A practical treatise on the diseases of the ear*, published in 1873 [23], 1874, and 1876. Nevertheless, he writes the same text in the three editions: “The mastoid should be perforated in the case of a suppuration of long standing, with frequent and painful exacerbation. The operation may now be sure to be fairly established, and is frequently undertaken, it having been performed by Follin, Schwartze […] and by myself since 1859.”

No mention of SEP is found in the few other listed otological textbooks published in 1873: *The causes and treatment of deafness; being a manual of aural surgery *by the British James Keene [24]; *Diseases of the ear* by the American A.D. Williams [25]; *Deafness and diseases of the ear: The causes and treatment* by the British John Pyne Pennefather [26], and *Lectures on diseases and injuries of the ear* by the British William Bartlett Dalby [27]. The French Jean-Pierre Bonnafont also does not mention SEP in the second edition of his 1873 *Traité théorique et pratique des maladies de l’oreille*, in which he notably writes about the trepanation of the mastoid process: “Empiricism alone directed the hand of the surgeons; and, just as they had no data before the operation, they were also very surprised by the few successes they obtained from it; because nowhere is there any mention of cases where the operation was carried out with a chance of success” [28].

### Reception of SEP in the medical and otological literature in 1874

In the 1874 *Archiv für Ohrenheilkunde*, there are no publications related to SEP. In the *Monatsschrift für Ohrenheilkunde*, however, the two last parts of Bezold’s articles are found, as already mentioned above. Buck’s publications form 1873 are also largely discussed [29, 30]. In the first issue of the 1874 *Archives of Ophthalmology and Otology*, the American Clarence John Blake writes: “In relation to the point to be chosen for perforating the mastoid, Dr. Bezold, following the descriptions given by Schwartze and Eysell, has made further examination into the anatomical proportions of the mastoid process with especial reference to this operation” [31]. This article is not printed in the German edition. In some 1874 general medical journals there is mention of SEP, such as in the *Centralblatt für die medicinischen Wissenschaften *(*Central Pamphlet for Medical Sciences*; [32]) in the form of an abstract (with Schwartze and Eysell as co-authors); in the *Berliner Klinische Wochenschrift *(*Berlin Clinical Weekly Journal*; [33]), in reference; and in the *Journal de Médecine, de Chirurgie et de Pharmacologie *(*Journal of Medicine, Surgery and Pharmacology*; [34]) as a comment with the description of the surgical technique with the chisels and gouges.

The German Carl Weitz completed his 1874 thesis about the surgical opening of the mastoid process with: “I would like to take this opportunity to express my sincere thanks to my highly esteemed teacher, Professor Dr. Schwartze, for the kind support he provided me with during the preparation of this work” [35].

In the Supplement written by the British James Hinton of the 1874 French edition of the textbook *Diseases of the ear* by the British Joseph Toynbee [36], SEP is shortly mentioned as a “compelled memory” on the opportunity to open the mastoid process. Interestingly, Hinton does not mention it in his own textbook published at the same time, *The question of aural surgery *[37]. No mention of SEP is found in the second edition of *Lectures on aural catarrh* by the Scottish Peter Allen [38].

### Reception of SEP in the medical and otological literature in 1875

In an issue of the *Archiv für Ohrenheilkunde*, Eysell commented on Bezold’s series of articles about the mastoid process published between 1873 and 1874 as mentioned before [19]. In none of the 1875 issues of the *Monatsschrift für Ohrenheilkunde*, nor in the *Annales des maladies de l’oreille, *are contributions related to SEP found. The *Archiv für Augen- und Ohrenheilkunde* did not publish a volume in 1875 (volume IV was published in 1874 and volume V in 1876). Also, SEP is not mentioned in the *Annales des maladies de l’oreille et du larynx* in 1875.

**Fig. 5:**
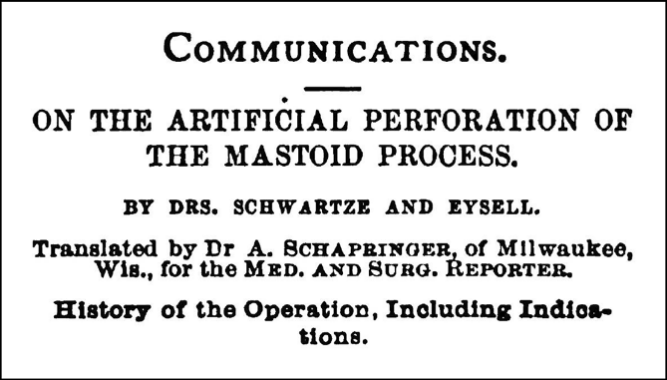
Reprint of the title of the article by Alois Schrapinger in the *Medical and Surgical Reporter* with a partial translation of the original publication by Hermann Schwartze and Adolf Eysell

On January 30, 1875, in the *Medical and Surgical Reporter*, the American Alois Schapringer partially translated SEP into English under the title “On the artificial perforation of the mastoid process” ([39]; Fig. 5). “We are firmly convinced that by certain improvements on the methods of operating […] the results will become more satisfactory than they have been.”

The German Wilhelm Kramer, in his second revised very critical edition *Die Ohrenheilkunde der letzten 50 Jahre *(*Otology in the last 50 years*), writes about the artificial perforation of the mastoid process: “From these considerations it appears undoubted that the artificial perforation of the eardrum is currently in the same dismal position as its inventor placed it. For the time being it is still a purely luxury item for ear doctors who like to write and operate. Almost the same applies to the one brought back to life by Schwartze artificial opening of mastoid process […] Under these very unsatisfactory circumstances one can only attribute very little value to Schwartze’s present work” [40]. In the first edition from 1873, Kramer does not mention SEP. The German Julius Erhard also does not mention SEP in his textbook from 1875 *Vorträge über die Krankheiten des Ohres *(*Lectures on the Diseases of the Ear*; [41]).

In the 1875 volume XXI of the *Nouveau dictionnaire de médecine et de chirurgie pratique *(*New practical dictionary of medicine and surgery*), there is: “Very recently, Schwartze and Eysell, through the analytical study of a large number of facts, demonstrated all the advantages that can be obtained from mastoid perforation” [42].

## Discussion

The implementation of a new, supposedly relevant, medical procedure or technique typically involves a dynamic, evolving, and structured process to ensure that the information is disseminated, correctly evaluated, and efficiently integrated into clinical practice. The publication of a medical article in a well-reputed and referenced journal assures its reliability and initial dissemination, which is also related to the way the journal is nationally and internationally distributed and how easily it is accessible. The period chosen for this study is too short to have a complete overview of the evolution of SEP in the medical literature and in clinical practice. Nevertheless, it is long enough to provide a first appreciation of its implementation. In 1873, only three otolaryngology journals existed, the oldest and most well-known being the *Archiv für Ohrenheilkunde *[43], the journal in which SEP was published. It was only sent by mail and was usually accessible through university medical libraries or personal subscription, which assured a good dissemination of SEP among the target group of medical practitioners in the field of otology. Good dissemination was also associated with the possible compilation and evaluation of the article by other journals, albeit not necessarily specialized journals. As a result, the publication became known to many more interested persons, and it was rapidly discussed in the English, German, and French otological and general medical literature in the second part of 1873. In 1874 and 1875, SEP was less mentioned, at least in the journals studied here. However, its partial translation into English in 1875 in a general medical journal is quite unusual for such a specific subject, and certainly demonstrates the importance of SEP. This fact is probably explained by a phase of larger evaluation, which is time consuming.

This journalistic dissemination was completed by the presentation of the paper at medical congresses and medical societies, by the author(s) of the paper or by other specialists having reviewed and integrated it in their clinical practice. Thus, SEP was discussed at least in one otological meeting in 1873. This usually introduces a more direct and open discussion with the participants and can improve the quality of the feedback loop, leading to modification and refinement of the results or procedures presented in the publication.

Another step is the integration into textbooks, usually edited for compiling and sharing more complete knowledge and continuing medical education, as is the case for SEP in at least one otological textbook as early as in 1873. As very few otological textbooks were published in 1874 and 1875, it is difficult to appreciate the implementation of SEP in this kind of support in such a short timeframe. Still, some comments appeared, notably in an 1875 textbook.

## Conclusion

The princeps paper *Ueber die künstliche Eröffnung des Warzenfortsatzes* by Hermann Schwartze and Adolf Eysell in 1873 was rapidly disseminated in the medical literature for nearly 1 year, and then entered a phase of evaluation followed by a phase of extension and implementation, later demonstrated by its definitive place in the history of surgery of the mastoid process. Commemorating its 150th jubilee is a good way to pay tribute to its two authors, Hermann Schwartze and Adolf Eysell.

## Declarations

For this article no studies with human participants or animals were performed by any of the authors. All studies mentioned were in accordance with the ethical standards indicated in each case.
